# Pathologic complete response to chemoimmunotherapy of an advanced gastric cancer patient with high PD-L1 expression, dMMR, and unique gut microbiota composition: A case report

**DOI:** 10.3389/fonc.2023.1150931

**Published:** 2023-03-16

**Authors:** Hongpeng Jiang, Junyun Wang, Wei Deng

**Affiliations:** ^1^ Department of General Surgery, Beijing Friendship Hospital, Capital Medical University & National Clinical Research Center for Digestive Diseases, Beijing, China; ^2^ CAS Key Laboratory of Genome Sciences and Information, Beijing Institute of Genomics, Chinese Academy of Sciences/China National Center for Bioinformation, Beijing, China

**Keywords:** camrelizumab, conversion therapy, Mismatch-repair deficiency, pathologic complete response, advanced gastric cancer

## Abstract

**Background:**

Advanced gastric cancer (AGC) is a malignant disease with limited therapeutic options and a poor prognosis. Recently, immune checkpoint inhibitors (ICIs), represented by inhibitors of programmed cell death 1 (PD-1)/programmed death-ligand 1 (PD-L1), have emerged as a potential gastric cancer (GC) therapy.

**Case presentation:**

This case study aimed to reveal the tumor response to neoadjuvant chemotherapy combined with camrelizumab in a patient with AGC based on the characteristics of the clinical pathology, genomics variation, and gut microbiome. Samples from a 59-year-old male patient diagnosed with locally advanced unresectable GC (cT4bN2M0, high grade) presenting PD-L1-positive, deficient mismatch repair (dMMR), and highly specific gut microbiota enrichment were subjected to target region sequencing, metagenomic sequencing, and immunohistochemistry staining. The patient received neoadjuvant therapy, including camrelizumab, apatinib, S-1, and abraxane, which eventually promoted dramatic tumor shrinkage without serious adverse effects and allowed subsequent radical gastrectomy and lymphadenectomy. Finally, the patient achieved pathologic complete response (pCR), and the recurrence-free survival time was 19 months at the last follow-up in April 2021.

**Conclusions:**

The patient with PD-L1-positive, dMMR, and a highly specific gut microbiota enrichment exhibited a pCR to neoadjuvant chemoimmunotherapy.

## Introduction

Gastric cancer (GC) is the sixth most frequently diagnosed malignant tumor worldwide, and it is one of the major causes of malignant disease morbidity and mortality in China (1, 2). Despite major advances in GC therapy in recent years, advanced GC (AGC) has remained a challenge in clinical practice. Chemoimmunotherapy is a more recent development in managing patients with advanced malignancy, including GC (3, 4). While The use of immune checkpoint inhibitors (ICIs) in conjunction with neoadjuvant chemotherapy to treat AGC is still debatable.

The validated checkpoint inhibitor response predictors include programmed death ligand-1 (PD-L1) expression, the tumor mutational burden (TMB), microsatellite status, and gut microbiota (5). Today, it is common practice to assess PD-L1 expression and deficient mismatch repair (dMMR) to assess the viability of immunotherapy, particularly for colorectal cancer. The diversity of the microbiome can also influence how the ICI responds (6). This case study involves a patient diagnosed with unresectable AGC with high PD-L1 expression (a tumor proportion score (TPS) of 5% and a combined positive score (CPS) of 80-90) and dMMR in the tumor tissues. This patient achieved pathologic complete response (pCR) after treatment with camrelizumab combined with apatinib, S-1, and abraxane.

## Case presentation

**Figure 1:**
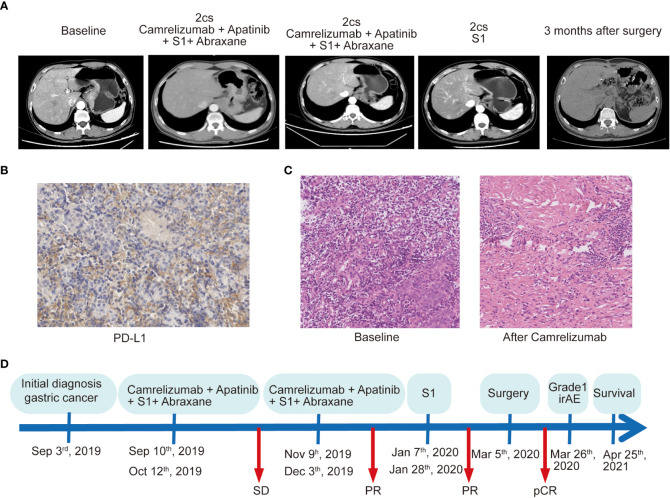
The treatment process and clinical outcome of the patient who achieved a pathologic complete response to conversion therapy. **(A)** Computed tomography scan shows the different stages of the primary lesions over time. **(B)** PD-L1 assay of the primary lesion on needle biopsy by immunohistochemical staining with DAKO 22C3 antibody. Magnification, ×200. **(C)** Pathological results of the primary specimens at baseline and after treatment with chemoimmunotherapy. Magnification, ×200. **(D)** Illustrating the timeline of the treatment course of the patient.

A 59-year-old male presented with epigastric pain and difficulty swallowing and was referred to our outpatient department. He had smoked for 40 years, had never taken medication, and occasionally drank alcohol. The physical examination was unremarkable. The serum values of the tumor markers, such as a-fetoprotein, carcinoembryonic antigen, and carbohydrate antigen 19-9, were almost normal. An abdominal computed tomography (CT) scan showed the circumferential wall of the lower section of the esophagus, cardiac and corpus thickening in the portal phase, and delayed enhancement of the thickening esophagus and stomach wall and perigastric lymph nodes (**Figure 1A**). Positron emission tomography (PET) confirmed 2-fluoro-2-deoxy-D-glucose (FDG)-avidity in the lower section of the esophagus, cardiac and corpus, and perigastric lymph nodes at stations 1, 3, 7, 9, 11, and 16. Subsequent endoscopy at our center revealed stenosis of the lower esophageal cavity, elevated circumferential lesions of the gastric body with involvement of the cardia, and biopsy-proven adenocarcinoma. A multidisciplinary team debate recommended that the patient start out with conversion therapy because the tumor was borderline resectable.

To assess a 620-gene panel of genomic change, a tissue sample acquired during an endoscopy was subjected to next-generation sequencing (NGS) with the patient’s permission (GloriousMed Clinical Laboratory Co., Shanghai, China), while the PD-L1 expression was also detected *via* immunohistochemistry (IHC). Genomic testing identified somatic mutations, including KMT2D Q3612del, FLCN F157del, CASP8 F414C, and LATS2 G223R, while the germline mutations of the DNA damage repair pathway genes included BLM S801T, MLH3 T1348I, MSH6, K646R, and ROS1 M1340T. No mutations were detected in TP53, CDH1, or ERBB2 (**Table 1**). The tumor mutational burden (TMB) was 2.25 mut/Mb, defined as TMB-low. The IHC results showed that the PD-L1 CPS of the tissue sample was 80-90 and the PD-L1 TPS was about 5%, indicating high PD-L1 expression (**Figure 1B**). Moreover, shotgun sequencing was done to determine the gut microbiota makeup for assessing the efficacy of immunotherapy. We observed an overrepresentation of specific strains, such as *Akkermansia muciniphila*, *Bifidobacterium longum*, *Odoribacter*, and *Veillonella* (**Table 2**). Chemoimmunotherapy was applied in response to these findings after the patient gave informed consent. After four-cycle treatment with camrelizumab (200 mg, day 1, i.v.), apatinib (250 mg, daily, oral), S-1 (80 mg/day, days1-14, oral), and abraxane (200 mg, days 1 and 8, i.v.), CT scans in January 2020 showed lesion shrinkage in the esophagus, stomach, and perigastric lymph nodes (**Figure 1A**). Next, the patient received S-1 monotherapy for two cycles between January 2020 and March 2020 due to personal reasons. CT scans in March 2020 revealed effective tumor downsizing in the esophagus, stomach, and perigastric lymph nodes.

**Table 1:** Genetic mutations of cancer-related pathways in the gastric cancer tissues obtained by endoscopy.

Pathway	Gene	AA change	Variant classification	Germline or somatic mutation	Frequency (%)
DDR pathway	*BLM*	p.S801T	Nonsynonymous mutation	Germline	49.87
	*MLH3*	p.T1348I	Nonsynonymous mutation	Germline	48.68
	*MSH6*	p.K646R	Nonsynonymous mutation	Germline	48.45
	*TPX2*	p.G723D	Nonsynonymous mutation	Germline	44.50
RTK/RAS pathway	*ROS1*	p.M1340T	Nonsynonymous mutation	Germline	48.63
Notch pathway	*NCOR2*	p.Q508_Q510del	Nonframeshift insertion	Germline	43.00
Histone Modifications	*KMT2D*	p.Q3612del	In frame deletion	Somatic	5.90
mTOR pathway	*FLCN*	p.F157del	In frame deletion	Somatic	5.70
Hippo pathway	*LATS2*	p.G223R	Missense mutation	Somatic	5.50
Toll-like receptor pathway	*CASP8*	p.F414C	Missense mutation	Somatic	4.90
Calcium pathway	*GRIN2A*	p.N447K	Nonsynonymous mutation	Germline	49.48

**Table 2:** Gut microbiota possibly related to response to immune checkpoint inhibitors for this patient.

Species	Enrichment abundance
Faecalibacterium prausnitzii	0.058406643
Odoribacter splanchnicus	0.025275101
Collinsella aerofaciens	0.005032293
Veillonella parvula	0.003084175
Akkermansia muciniphila	0.001871486
Clostridiales bacterium CCUnclassified10	0.001641917
Veillonella atypica	0.001524239
Veillonella dispar	0.0010913
Bifidobacterium breve	0.000636746
Enterococcus faecium	0.000125646
Bifidobacterium pseudolongum1	1.67019E-05
Lactobacillus johnsonii	1.63744E-05
Olsenella sp. GAM18	6.12403E-05

Due to significant tumor shrinkage, the patient received curative resection in March 2020. The anterior gastric wall, the cardia at the lesser curvature, and the gastric fundus were all palpable with tough masses and numerous swollen lymph nodes surrounding the stomach, as revealed by intraoperative exploration. The tumor proliferated upward to above the esophageal hiatus and the left diaphragm. Eventually, the lower esophagus, entire stomach, part of the left diaphragm, and perigastric lymph nodes were surgically removed. Specimens were then sent for further pathological evaluation. No cancer cells or positive lymph nodes were found in any of the resected specimens, indicating that the patient achieved pCR after conversion therapy. The histological assessement revealed severe necrosis and fibrosis. (**Figure 1C**). The entire course of the clinical treatment is illustrated in **Figure 1D**. The patient continued to exhibit disease stability at the time of the last follow-up in April 2021 and achieved pCR lasting more than 19 months.

## Discussion

Anti-PD-1/PD-L1 inhibitors have proven superior to standard therapy when used to treat a variety of advanced solid tumors (7). Although anti-PD-1/PD-L1 inhibitors and chemotherapy have been approved for the first-line treatment of AGC, there is presently no accepted standard of care for some locally advanced patients that may benefit from surgery (8). For patient treatment, research is currently being done to determine any potential advantages of combining chemotherapy with anti-PD-1/PD-L1 inhibitors. The current attempt to treat our patient with unresectable AGC using camrelizumab in combination with apatinib, S-1, and abraxane has been quite successful. We hypothesize that the positive outcome of our patient can be attributed to high PD-L1 expression and dMMR status given the relatively high levels of CPS and *MSH6* mutation in the tissue samples.

As a potential reference, a randomized phase 3 clinical trial (also known as KEYNOTE-062) investigated the efficacy and safety of pembrolizumab for AGC or gastroesophageal junction cancer (9). In a population with CPS ≥ 10, patients receiving pembrolizumab showed a significantly favorable response, with longer survival times than those receiving chemotherapy (cisplatin and fluorouracil or capecitabine), which showed that immunotherapy with ICIs against PD-1/PD-L1 should be revisited and improved as the first-line AGC treatment strategy (9). Patients receiving pembrolizumab outlived those receiving chemotherapy for AGC with dMMR, according to a comparison of their survival rates (9). Coincidentally, researchers treated 12 patients with dMMR rectal cancer using dostarimab, a monoclonal antibody inhibitor of PD-1, with all patients achieving pCR (10). Extensive studies that have been conducted recently have shown how crucial the combination of immunotherapy and chemotherapy is for first-line AGC treatment (8, 11). The CheckMate-649 trial, the most significant clinical trial for AGC thus far, revealed that combining nivolumab and chemotherapy prolonged progression-free and overall survival of patients with CPS ≥ 5, compared with chemotherapy (8). Similar to this, the Attraction-4 trial, which focused on the Asian population particularly, confirmed the efficacy of nivolumab and chemotherapy when used together as a first-line AGC treatment. In terms of progression-free survival and the objective response percentage of patients, the combination was superior to chemotherapy alone (11). The early findings of the current studies offered strong support for the use of chemoimmunotherapy as a first-line therapy for AGC.

Based on the results of the DELIVER trial, it is likely that the composition of the gut microbiome influences the effectiveness of anti-PD-1 therapy, while specific bacterial genera in the gut microbiome are positively correlated with patient outcomes in AGC (12). *Akkermansia muciniphila* and *Bifidobacterium longum* have been found to promote anticancer efficacy (13). Microbiota analyses revealed a high abundance of *Akkermansia muciniphila* and *Bifidobacterium longum* in the fecal samples of our patient, demonstrating that the clinical effectiveness of immunotherapy was more likely to be improved by these particular strains. Further research has shown that fecal microbiota transplant can increase the treatment response in advanced melanoma by overcoming anti-PD-1 resistance (14, 15). These unexpected results have significant implications for treating patients with advanced cancer, even though the sample sizes used in the current research are not yet sufficiently scalable. There are various current initiatives to validate these findings in AGC patients.

## Limitations

The chemotherapy backbone utilized here is not used in other parts of the world and is approved in China.

## Conclusion

A Chinese patient with unresectable AGC and high PD-L1 and dMMR expression who responded favorably to the combined therapy is the subject of this case report. The development of future chemoimmunotherapy techniques for AGC is significantly influenced by the results of targeted NGS of a panel of hundreds of cancer genes.

## Data availability statement

The original contributions presented in the study are included in the article/supplementary material. Further inquiries can be directed to the corresponding author.

## Ethics statement

The studies involving human participants were reviewed and approved by the ethics committee of Beijing Friendship Hospital. The patients/participants provided their written informed consent to participate in this study.

## Author contributions

HJ: Acquisition of Data; Analysis and Interpretation of Data; Drafting of the Manuscript; Critical Revision of the Manuscript for Important Intellectual Content; Obtained Funding. JW: Technical, or Material Support. WD: Study Concept and Design; Acquisition of Data; Aanalysis and Interpretation of Data; Critical Revision of the Manuscript for Important Intellectual Content; Technical, or Material Support; Study Supervision. All authors contributed to the article and approved the submitted version.
